# Reinventing magnetic resonance imaging for accessible healthcare: Whole‐body imaging at 0.05 Tesla

**DOI:** 10.1002/ctm2.70071

**Published:** 2024-10-23

**Authors:** Ed X. Wu, Xiaoyuan Feng

**Affiliations:** ^1^ Laboratory of Biomedical Imaging and Signal Processing, The University of Hong Kong Pokfulam China; ^2^ Department of Electrical and Electronic Engineering The University of Hong Kong Pokfulam China; ^3^ Department of Radiology Huashan Hospital, Fudan University Shanghai China

1

Magnetic resonance imaging (MRI) technology has revolutionized the field of medical imaging by providing a non‐invasive, non‐ionizing and quantitative approach to visualizing different tissue types and assessing their structural and physiological integrity. Despite its importance and five decades of engineering development, the accessibility of MRI is low and extremely inhomogeneous around the world. This is due to the high cost and complex infrastructure requirements of existing high‐field superconducting MRI scanners, which limit their availability in low and middle‐income countries, and exclude their easy access in many healthcare facilities such as neurology clinics, trauma centres, surgical suites, neonatal/pediatric centres and community clinics. We and others have made intensive efforts in recent years to engineer low‐cost and shielding‐free MRI scanners for brain imaging at ultra‐low‐field (ULF) strengths (<0.1 T).^1^, ^2^, ^3^, ^4^, ^5^ However, these developments are limited to brain and extremity imaging and their image quality is generally poor.

Recently we have developed a compact, low‐power and highly simplified ULF MRI scanner that enables whole‐body imaging with high image quality via computing.^6^ In this work, we designed and prototyped a cost‐effective whole‐body 0.05 T MRI scanner that operates on a standard AC wall power outlet without any radiofrequency (RF) or magnetic shielding cages. The system utilized a compact 0.05 T permanent neodymium ferrite boron magnet with a double‐plate structure and required no RF shielding cages (Figure 1A). To address electromagnetic interference (EMI) during scanning, we deployed active EMI sensing during scanning and deep learning direct signal prediction (Deep‐DSP) strategy to retrospectively predict EMI‐free MR signals prior to image reconstruction^5^, ^7^, ^8^ (Figure 1B). We implemented and experimentally optimized the commonly used imaging protocols using phantoms and volunteers, keeping scan time at 8 min or less for each protocol. Our Deep‐DSP strategy showed superior performance compared to other EMI reduction methods. Using traditional Fourier image reconstruction, we were able to image various anatomical structures with different MRI contrasts, including the brain, spine, abdomen, lung, extremities and heart (Figure 2A). We were also able to estimate cardiac function and visualize major vessels in the neck using time‐of‐flight magnetic resonance angiography without any exogenous contrast agent. However, these images exhibited a high level of noise and artefacts due to a drastically reduced MR signal at 0.05 T versus the standard 3 T. To address this challenge, we developed novel data‐driven deep‐learning image reconstruction methods to significantly advance ULF MRI image quality. We formulated a 3D partial Fourier super‐resolution (PF‐SR) strategy^9^, ^10^ that integrates image reconstruction and super‐resolution. By learning from high‐field MRI datasets, the PF‐SR reconstruction approach improved image quality by suppressing artefacts and noise and increasing spatial resolution. The PF‐SR method enhanced the clarity and visualization of the brain, spine, abdomen and knee (Figure 2B). These PF‐SR results demonstrate the unprecedented capability of deep learning PF‐SR image reconstruction in advancing ULF MRI.

**FIGURE 1:**
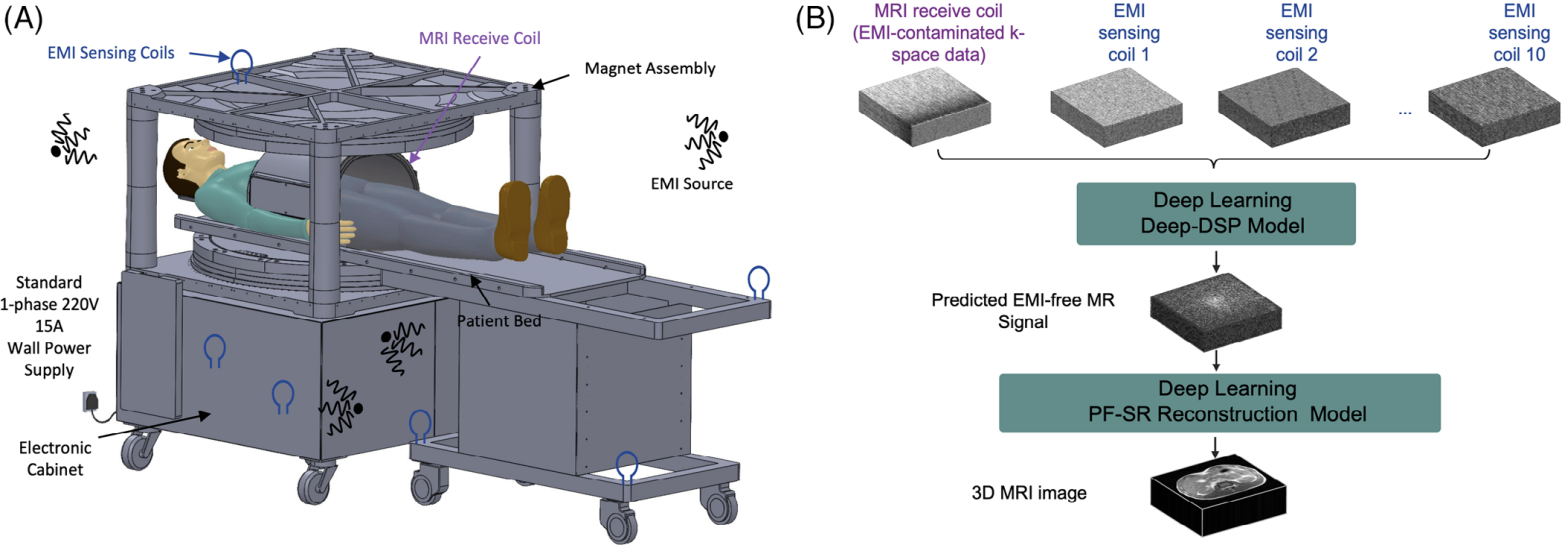
(A) A low‐cost, compact and computing‐powered 0.05 T magnetic resonance imaging (MRI) scanner prototype for whole‐body imaging that operates on a standard AC wall power outlet without any RF or magnetic shielding. The scanner is equipped with a number of small electromagnetic interference (EMI) sensing coils that actively sense both external and internal EMI signals during scanning in the absence of the conventional RF shielding enclosure. (B) After each scan, EMI‐free MR signals are directly predicted using the deep learning direct signal prediction (Deep‐DSP) method,^5^, ^7^, ^8^ a recently developed deep learning approach that simultaneously acquires signals by both the MRI receive coils and EMI sensing coils and retrospectively predicts EMI‐free MR signals. High‐quality images are then reconstructed from the EMI‐free MR signals using the recently developed deep learning 3D partial Fourier super‐resolution (PF‐SR) models.^9^, ^10^

**FIGURE 2:**
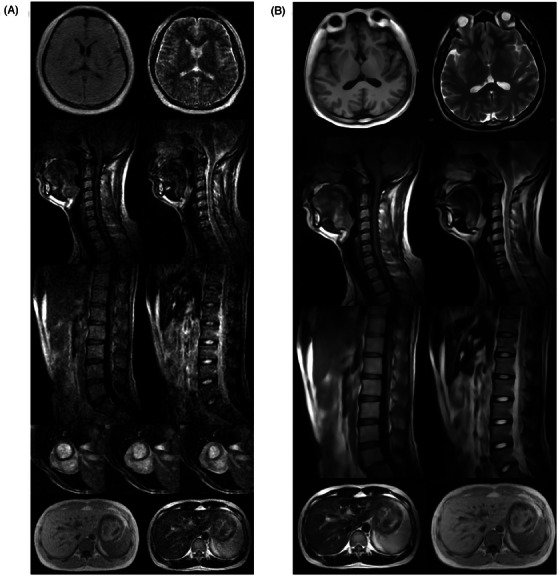
(A) 0.05 T images of different anatomical structures reconstructed with traditional Fourier methods. (B) 0.05 T images reconstructed using the data‐driven deep learning partial Fourier super‐resolution (PF‐SR) methods, leveraging large‐scale high‐field magnetic resonance imaging (MRI) data to generate high‐resolution images.

The computing‐powered whole‐body ULF MRI technology demonstrated in this study has the potential to complement existing high‐performance high‐field clinical MRI by providing a more affordable and accessible option. ULF MRI offers several distinct advantages, including an open scanning environment, less acoustic noise, low sensitivity to metallic implants, less image susceptibility artefacts at air/tissue interfaces and an extremely low RF‐specific absorption rate. ULF MRI also provides a more comfortable experience for patients and is advantageous in the presence of metal implants or fragments. Additionally, imaging at ULF enables more time‐efficient data acquisition protocols due to faster longitudinal magnetization recovery and slower transverse magnetization decay.^5^ The ULF MRI scanner is inherently affordable and easy to operate. Though not shown in this study, we envision that the scanner operation could be easily automated via a simple, smart user interface, such as a single‐button scan with automatic imaging localization, requiring no special training for users. Such simplified yet intelligent MRI can be especially well‐suited for healthcare in low‐ and middle‐income countries, as well as in developed countries where it can broaden MRI usage beyond traditional radiology departments. By offering low‐cost screening, diagnosis, monitoring and image‐guided intervention, healthcare providers can bring imaging capabilities closer to patients, ultimately enhancing patient care and outcomes. Whole‐body MRI is also valuable in detecting and characterizing various types of cancers, such as liver, pancreatic, prostate, breast and colorectal cancer, and can potentially be used to characterize diffuse liver diseases using MRI techniques like magnetic resonance elastography and liver fat quantification on the whole‐body ULF MRI scanner. Future studies are needed to advance ULF MRI technology and evaluate its clinical efficacy, particularly in neurology clinics, trauma centres, surgical suites and neonatal/pediatric centres.

Achieving optimal ULF MRI diagnostic quality (in terms of image signal‐to‐noise ratio, contrast and spatial resolution) within a reasonable scan time remains a challenge. Deep learning can further advance ULF MRI image formation through more powerful data‐driven image deep learning architectures. Future ULF MRI hardware development can also focus on more sensitive MRI receive coils and/or more intelligent signal reception approaches at the RF megahertz range through design or material innovation. As an easy‐to‐access imaging device, ULF MRI must function fully without an RF shielding enclosure. Although the Deep‐DSP approach can directly predict EMI‐free MR signals in the presence of very strong EMI signals, new and robust EMI elimination strategies need to be continuously developed to accommodate imaging scenarios with extremely strong and diverse EMI sources. Additionally, an ideal whole‐body ULF MRI scanner must be lightweight, and future development should explore novel magnet designs for lightweight and small footprints.

In summary, our study tackled the challenge of limited MRI accessibility by developing an affordable, simple and computing‐powered whole‐body 0.05 T MRI scanner.^6^ Our results in imaging various anatomical structures demonstrated the versatility of ULF MRI, while the use of 3D deep learning reconstruction significantly improved the ULF image quality by leveraging high‐field MRI data and computing power. Our compact ULF scanner can operate from a standard AC wall power outlet without the need for RF or magnetic shielding. This makes it a practical and ubiquitous point‐of‐care imaging device in various healthcare settings, similar to ultrasound scanners. Looking ahead, the ever‐expanding computing power and large‐scale data will likely lead to the creation of a new class of MRI scanners. These affordable, patient‐centric and portable scanners will address unmet clinical needs in various healthcare settings worldwide, further revolutionizing the field of medical imaging.

## CONFLICT OF INTEREST STATEMENT

The authors declare no conflict of interest.
